# Novel Mutations and Mutation Combinations of* TMPRSS3* Cause Various Phenotypes in One Chinese Family with Autosomal Recessive Hearing Impairment

**DOI:** 10.1155/2017/4707315

**Published:** 2017-01-29

**Authors:** Xue Gao, Yong-Yi Yuan, Guo-Jian Wang, Jin-Cao Xu, Yu Su, Xi Lin, Pu Dai

**Affiliations:** ^1^Department of Otolaryngology, The General Hospital of the PLA Rocket Force, No. 16 Xinwai Dajie, Beijing 100088, China; ^2^Department of Otolaryngology, Head and Neck Surgery, PLA General Hospital, No. 28 Fuxing Road, Beijing 100853, China; ^3^Department of Otolaryngology, Emory University School of Medicine, 615 Michael Street, Whitehead Biomedical Research Bldg, Rm No. 543, Atlanta, GA 30322, USA

## Abstract

Autosomal recessive hearing impairment with postlingual onset is rare. Exceptions are caused by mutations in the* TMPRSS3* gene, which can lead to prelingual (DFNB10) as well as postlingual deafness (DFNB8).* TMPRSS3* mutations can be classified as mild or severe, and the phenotype is dependent on the combination of* TMPRSS3* mutations. The combination of two severe mutations leads to profound hearing impairment with a prelingual onset, whereas severe mutations in combination with milder* TMPRSS3* mutations lead to a milder phenotype with postlingual onset. We characterized a Chinese family (number FH1523) with not only prelingual but also postlingual hearing impairment. Three mutations in* TMPRSS3*, one novel mutation c.36delC [p.(Phe13Serfs⁎12)], and two previously reported pathogenic mutations, c.916G>A (p.Ala306Thr) and c.316C>T (p.Arg106Cys), were identified. Compound heterozygous mutations of p.(Phe13Serfs⁎12) and p.Ala306Thr manifest as prelingual, profound hearing impairment in the patient (IV: 1), whereas the combination of p.Arg106Cys and p.Ala306Thr manifests as postlingual, milder hearing impairment in the patient (II: 2, II: 3, II: 5), suggesting that p.Arg106Cys mutation has a milder effect than p.(Phe13Serfs⁎12). We concluded that different combinations of* TMPRSS3* mutations led to different hearing impairment phenotypes (DFNB8/DFNB10) in this family.

## 1. Introduction

Hearing impairment is the most common sensory disorder. Identifying the genetic basis of deafness provides important information for the diagnosis, intervention, and treatment of this condition. Nonsyndromic hearing impairment, however, is extremely genetic heterogeneous. To date, more than 200 genetic loci have been mapped, and 100 deafness genes have been identified (http://hereditaryhearingloss.org/). Autosomal recessive nonsyndromic hearing impairment (ARNSHI) is the most common type and accounts for ~80% of cases of inherited hearing loss. A large majority of recessive mutations are associated with congenital, nonprogressive, and severe-to-profound hearing loss [1]. Exceptions were observed in the* TMPRSS3* gene, which was reported to be associated with two different phenotypes: DFNB10-associated hearing impairment has been reported to be prelingual (OMIM 605511), whereas DFNB8-associated hearing impairment is typically late onset and postlingual (OMIM 601072).* TMPRSS3* mutations can be classified as mild or severe, and the hearing phenotype is dependent on the combination of the two* TMPRSS3* mutant alleles [2]. The compound heterozygosity for a mild and severe mutations leads to postlingual hearing loss, whereas the combination of two severe mutations leads to profound hearing impairment with a prelingual onset [2]. The* TMPRSS3 *gene has been identified as the gene responsible for ARNSHI in several populations, including Asian, Mediterranean, and Caucasian populations [2–5].

This study identified one Chinese family with ARNSHI (number FH1523), in which affected individuals had two different hearing impairment phenotypes: prelingual and postlingual. We performed large-scale mutational screening of 129 known deafness-related genes and identified three disease-segregating mutations in the* TMPRSS3 *gene: two previously reported missense mutations, c.916G>A (p.Ala306Thr) and c.316C>T (p.Arg106Cys), and one novel mutation, c.36delC [p.(Phe13Serfs*∗*12)]. We concluded that different combinations of mutations led to different hearing impairment phenotypes in this family.

## 2.   Materials and Methods

### 2.1. Clinical Data

Family FH1523 is a four-generation Chinese family with prelingual and postlingual nonsyndromic sensorineural hearing loss. DNA samples were obtained from seven members of family FH1523 (II: 2, II: 3, II: 5, III: 2, III: 3, III: 4, and IV: 1) and 260 ethnicity-matched controls. A detailed medical history was obtained through a questionnaire. Computed tomography (CT) of the temporal bone was performed in the index patient (IV: 1). A physical examination, otoscopy, and pure tone audiometric examination (at frequencies from 125 to 8000 Hz) were performed to identify the phenotype. The hearing loss range was described depending on the pure tone audiometry (PTA) parameters: low frequency, 125–500 Hz; mid frequency, 1-2 kHz; and high frequency, 4–8 kHz. Prelingual and postlingual hearing loss was classified according to the onset of prominent hearing loss. Prelingual hearing loss is present before speech develops and usually begins before 3 years of age, whereas postlingual hearing loss occurs after the development of normal speech [6].

The index patient (IV: 1) underwent vestibular and ocular motor tests. The tests included evaluation of the vestibuloocular reflex using electronystagmography with computer analysis and saccadic, smooth pursuit, and horizontal optokinetic nystagmus responses. Vestibular stimulation comprised rotatory and caloric tests.

### 2.2. DNA Preparation

All genomic DNA was extracted from peripheral blood using a blood DNA extraction kit according to the manufacturer's instructions (TianGen, Beijing, China).

### 2.3. Deafness Gene Capture and Sequencing

Three prevalent deafness-associated genes,* GJB2*,* SLC26A4*, and mtDNA*12SrRNA*, were first screened for mutations in all participating cases and controls. Two affected members (II: 2 and IV: 1) and two unaffected members (III: 2 and III: 4) of family FH1523 were subjected to a gene panel containing 129 deafness genes. Capture and NGS of the coding exons and their flanking 100 bps for the 129 deafness genes (see Appendix table1 in Supplementary Material available online at https://doi.org/10.1155/2017/4707315) on a Illumina HiSeq2000 were performed by Otogenetics Corporation (Norcross, GA).

The details of the deafness gene capture, sequencing, and bioinformatics analysis methods have been described in detail previously [7]. According to the autosomal recessive pattern of inheritance, only variants that were homozygous or compound heterozygous in the affected siblings were selected as candidates.

### 2.4. Mutational Detection and Analysis of* TMPRSS3*

The segregation of the* TMPRSS3* c.916G>A (p.Ala306Thr), c.36delC [p.(Phe13Serfs*∗*12)], and c.316C>T (p.Arg106Cys) mutations was tested in seven family members (II: 2, II: 3, II: 5, III: 2, III: 3, III: 4, and IV: 1), including the four whose gDNA had been subjected to the 129 deafness-associated genes and next-generation sequencing (NGS) analysis, using polymerase chain reaction (PCR) (primer sequences available on request) followed by bidirectional Sanger sequencing of the amplified fragments (ABI 3100; Applied Biosystems). Sequence alterations were identified by alignment with the* TMPRSS3 *GenBank sequence (NM_024022.2, NP_076927.1) using the GeneTools software. In addition, a total of 260 ethnicity-matched controls with normal hearing underwent mutation screening by direct sequencing.

### 2.5. Multiple Sequence Alignment

Multiple sequence alignment was performed according to a HomoloGene program with default settings and the sequences* NP_001243246.1 (H. sapiens), XP_001105841.2 (M. mulatta), XP_001137100.3 (P. troglodytes), XP_001179855.1 (B. taurus), XP_853682.3 (C. lupus), NP_001157248.1 (M. musculus), NP_001101089.1 (R. norvegicus), XP_425558.3 (G. gallus), and XP_001340422.5 (D. rerio)*.

(http://www.ncbi.nlm.nih.gov/homologene?cmd=Retrieve&dopt=MultipleAlignment&list_uids=56985)

### 2.6. Model Building and Structure-Based Analysis

Three-dimensional (3D) modeling of the human wild type, p.Ala306Thr and p.Arg106Cys, was performed using SWISS-MODEL, an automated homology modeling program (http://swissmodel.expasy.org/workspace/). This study used the automatic modeling approach to apply the complete protein sequence of human TMPRSS3, including its 454 amino acids and its mutation, which are available in the NCBI GenBank (NP_001243246.1) in the FASTA format. Data obtained by the homology models were visualized using Swiss-PdbViewer 4.1.

## 3. Results

### 3.1. Family and Clinical Evaluations

**Figure 1:**
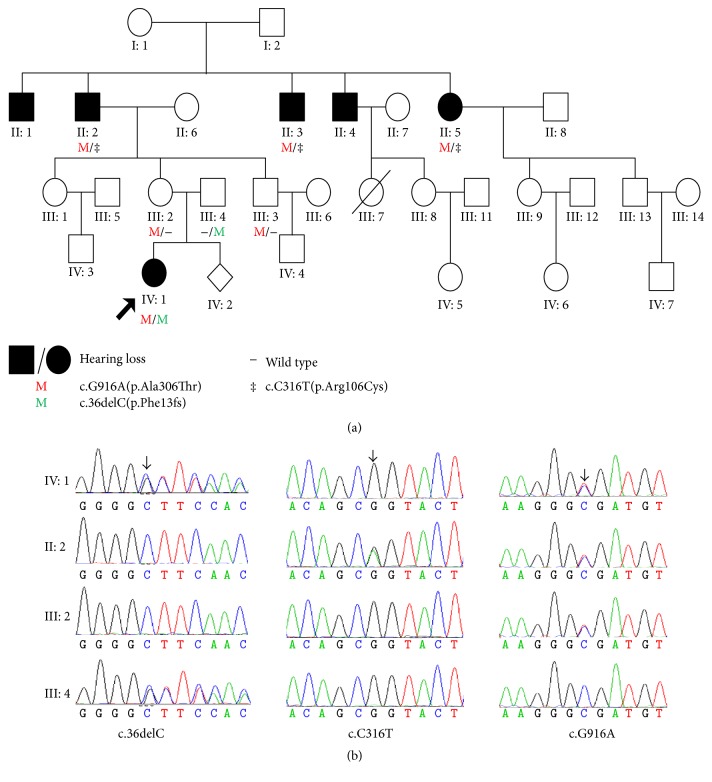
Pedigree of Chinese Family FH1523 with ARNSHI and mutation analysis of* TMPRSS3*. (a) The proband is indicated by an arrow. Subjects II: 2, IV: 1, III: 2, and III: 4 were tested by NGS. gDNA from II: 1 and II: 4 is not available. (b) DNA sequencing profile showing the c.916G>A, c.36delC, and c.316C>T mutations in* TMPRSS3*. Three variants cosegregated with the clinical phenotype.

**Figure 2:**
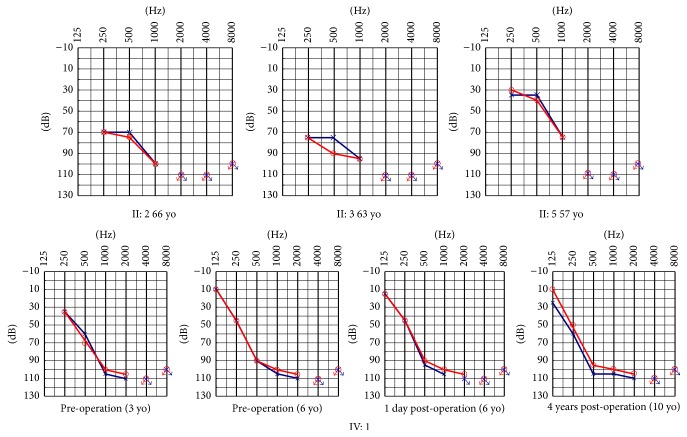
Audiogram showed bilateral sensorineural hearing impairment of affected subjects II: 2, II: 3, and II: 5 (upper panel) and IV: 1 (lower panel) (red, right ear; blue, left ear).

Family FH1523 is a four-generation Chinese family with ARNSHI (Figure 1) and includes six affected patients: II: 1 (male, 71 years old), II: 2 (male, 66 years old), II: 3 (male, 63 years old), II: 4 (male, 61 years old), II: 5 (female, 57 years old), and IV: 1 (female, 10 years old). For IV: 1, the index case, hearing impairment was severe and prelingual with failure of the newborn hearing test. Available audiograms showed slow progression from age 3 to 10 years. The hearing loss of IV: 1 was initially evaluated comprehensively at 3 years of age, with bilateral hearing impairment present with a ski-slop audiogram; the threshold of 250 Hz was 35 dBHL and that of 1 kHz to 8 kHz was over 100 dBHL. Although the threshold of 125 Hz was not tested, we predicted that it was lower than 10 dBHL according to the audiogram at 6 years of age. The hearing loss showed mild progression with annual threshold deteriorations of about 3-4 dBHL on average at 250 Hz and 7–10 dBHL on average at 500 Hz from 3 years to 6 years of age. She underwent right cochlear implantation at 6 years of age, and the residual hearing has been very well preserved for more than 4 years. A recent audiogram at 10 years of age showed that the threshold of 125 Hz was 10–25 dBHL and that of 250 Hz was 50–60 dBHL (Figure 2, lower panel).

For her affected grandfather (II: 4) and grandfather's four cousins (II: 1, II: 2, II: 3, and II: 5), hearing impairment was postlingual, late onset (in the second or third decades of life), and progressive according to memory description. Their hearing progressed gradually with advancing age. Their previous audiograms were absent due to living in the isolated village of China. They developed normal speech ability and now have oral communication capacities with the help of lip reading. They complained of having trouble detecting sharp voices since their 20s to 30s. We predicted that although low-frequency hearing was initially normal in the affected subjects of the second generation, their hearing would ultimately deteriorate at all frequencies. Flatter audiogram configurations were observed at ages older than 60 years (II: 2, II: 3), whereas the audiogram of II: 2 (57 years of age) was ski-sloped, and the threshold of 250 Hz was 30–35 dBHL, that of 500 Hz was 35–40 dBHL, that of 1 kHz was 75 dB, and that of 2 kHz to 4 kHz was greater than 100 dBHL (Figure 2, upper panel). For the affected subjects II: 1 and II: 4, gDNA and audiograms were unavailable.

Detailed vestibular analysis was performed in IV: 1 who did not complain about dizziness, vertigo, or imbalance. Vestibular tests revealed normal vestibular function via caloric tests. All position tests produced no nystagmus without vertigo sensation. Affected individuals did not have obvious delayed gross motor development. The physical examinations of all participating members revealed no signs of systemic illness or dysmorphic features. High-resolution computed tomography of the temporal bone in IV: 1 was normal, excluding inner ear malformations. This phenotype was consistent with that reported for DFNB8/10.

### 3.2. Targeted Deafness Gene Capture and Massively Paralleled Sequencing

We sequenced all the coding exons plus ~100 bp of the flanking intronic sequence of 129 deafness genes in two affected (II: 2, IV: 1) and two unaffected members of family FH1523 (III: 2, III: 4). Three variants leading to amino acid change were detected in* TMPRSS3*: c.916G>A (p.Ala306Thr) (rs181949335), c.36delC [p.(Phe13Serfs*∗*12)], and c.316C>T (p.Arg106Cys) (rs139805921). Of these, two variants, c.916G>A (p.Ala306Thr) and c.316C>T (p.Arg106Cys), were reported previously as a pathogenic mutation, whereas c.36delC [p.(Phe13Serfs*∗*12)] was novel and had not been found in the dbSNP databases.

### 3.3. Sanger Sequencing

Using Sanger sequencing, seven participating family members (four affected and three unaffected) in family FH1523 were genotyped to identify the mutations. Compound heterozygous p.Ala306Thr and p.Arg106Cys mutations of* TMPRSS3 *were identified in three affected family members of the second generation (II: 2, II: 3, and II: 5). Compound heterozygous p.(Phe13Serfs*∗*12) and p.Ala306Thr mutations of* TMPRSS3 *were found in one affected family member of the fourth generation (IV: 1). In addition,* TMPRSS3 *heterozygous variant p.Ala306Thr was found in two normal hearing family members (II: 2, II: 3), whereas heterozygous variant p.(Phe13Serfs*∗*12) was found in one normal hearing family member (III: 4) (Figure 1), which is consistent with autosomal recessive inheritance. None of the three mutations was found in 260 control individuals with normal hearing.

### 3.4. Correlation between Phenotype and Genotype

Because all the patients in family FH1523 had the p.Ala306Thr mutation in common, a comparison of the phenotypic effect of the p.Arg106Cys and the p.(Phe13Serfs*∗*12) was possible. The age at onset and severity of the hearing impairment in IV: 1 [p.Ala306Thr and p.(Phe13Serfs*∗*12)] were much earlier and worse, respectively, than those in II: 2, II: 3, and II: 5 (p.Ala306Thr and p.Arg106Cys). Comparison of the audiograms of IV: 1 (6 years of age) and II: 5 (57 years of age) revealed that II: 5 had a better hearing threshold at 250 Hz and 500 Hz. This result suggests that p.Arg106Cys mutation has a milder effect than p.(Phe13Serfs*∗*12).

**Figure 3:**
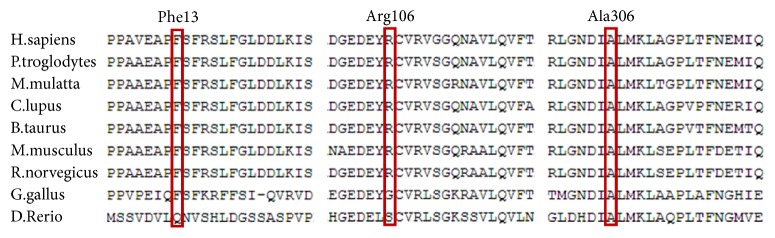
Conservation analysis of residues* TMPRSS3 *in family FH1523. Protein alignment showing conservation of residues TMPRSS3 Ala306, Phe13, and Arg106 across nine species. Three mutations all occurred at evolutionarily conserved amino acids (in red box).

Three amino acid substitutions occurred in evolutionarily conserved regions across different species (Figure 3) and were predicted to be damaging by SIFT, Polyphen2, and CADD.

### 3.5. Structure Modeling

The Ala306Thr molecular model covered the target sequence of TMPRSS3 (residues 217–451) and was constructed based on the crystal structure of FAB DX-2930 in complex with human plakallikrein (PDB ID: 4ogyB). The sequence identity between the target and template was 41.67%. Using Swiss-Pdb Viewer 4.1, the mutation was predicted to perturb the amino acid side chain due to the substitution of alanine to threonine at position 306. The wild-type alanine forms two salt bridges with threonine at position 254, whereas the mutant-type threonine forms an extra salt bridge with valine at position 291. The salt bridge differences will likely disturb the interaction made by the wild-type residue (Figure 5(a)).

The Arg106Cys molecular model covered the target sequence of TMPRSS3 (residues 71–109) and was constructed based on the crystal structure of C5B-6 (PDB ID: 4e0sB). The sequence identity between the target and template was 43.59%. Using Swiss-Pdb Viewer 4.1, the mutation showed that the wild-type and mutant amino acids differed in size. The mutant residue was smaller, a feature that might lead to the loss of interactions (Figure 5(b)).

These data, together with the clinical presentation of the affected siblings and consistent autosomal recessive inheritance of the mutations in the affected and unaffected members, indicate that the* TMPRSS3 *mutations, p.Ala306Thr, p.Arg106Cys, and p.(Phe13Serfs*∗*12), are the cause of hearing impairment in this family.

### 3.6. Cochlear Implantation

Individual IV: 1 underwent unilateral cochlear implantation (Cochlear, Freedom) at our hospital at the age of 6 years, and the electrodes were completely inserted into the right cochlea using the Advance-Off Stylet (AOS™) technique. The residual hearing of her right ear was very well preserved for more than 4 years (Figure 2(b)), and her listening and speech abilities were clearly improved after surgery. Our results indicate that ARNSHI patients with* TMPRSS3 *mutations are good candidates for residual hearing preservation by cochlear implantation, which is in accordance with the evaluation of cochlear implantation outcomes reported by Chung et al. [8].

## 4. Discussion 

Gene mutations associated with late-onset progressive disorders are most likely to result in less drastic changes in protein structure and function [8]. The progressive nature of these diseases could then be explained by a gradual increase in the ratio of damaged to normal proteins or by changes in protein levels [9].

**Figure 4:**
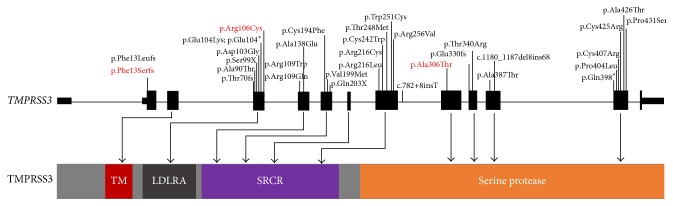
Genomic structure of* TMPRSS3* based on the open reading frame (NM_024022.2) containing 13 exons (black rectangles). The positions of 32* TMPRSS3* mutations are shown both at the gene (top) and at the protein level (bottom). The protein diagram depicts the predicted functional domains and sequence motifs. The mutations identified in this study are highlighted in red. TM, transmembrane domain; LDLRA, LDL receptor-like domain; SRCR, scavenger receptor cysteine-rich domain; serine protease, trypsin-like serine protease domain.

**Figure 5:**
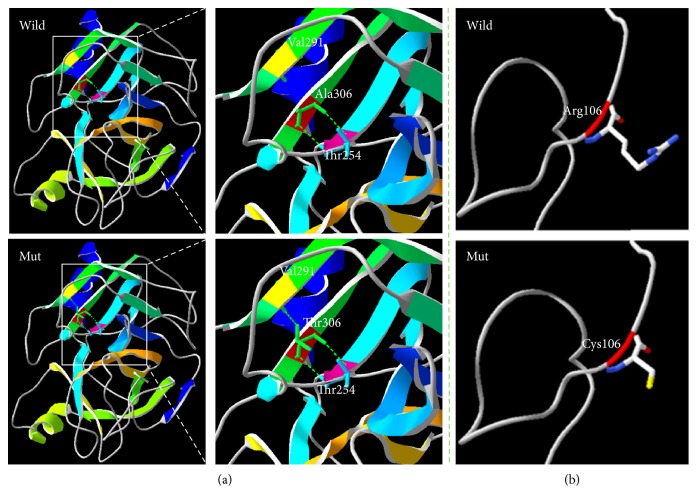
Molecular modeling of wild-type and mutant TMPRSS3. (a) The mutant protein Thr306 is predicted to have three hydrogen bonds and add one hydrogen bond compared to the wild-type protein. (b) Arg106 lies within a loop. The wild-type protein has a longer side chain than the mutant protein.

*TMPRSS3 *has 13 exons and encodes a protein that belongs to the serine protease family. The protein consists of 453 amino acids and contains a serine protease domain, a transmembrane domain (TM) (49–69 aa), an LDLRA domain (74–107 aa), a scavenger receptor cysteine-rich domain (SRCR) (112–211 aa), and a trypsin-like serine protease domain (216–446 aa) (NP_001243246.1) (Figure 4). RNA in situ hybridization on rat and mouse cochlea revealed that Tmprss3 is expressed in the cell bodies of the spiral ganglion neurons, inner hair cells, supporting cells, and stria vascularis of the cochlea [10]. TMPRSS3 plays an important role in activating the ENaC sodium channel and maintaining a low Na^+^ concentration in the endolymph of the inner ear [10–12]. ENaC is a sodium channel known to be regulated by serine protease activity [13].

TMPRSS3 is very important in the auditory system and was also identified as a tumor-associated gene that is overexpressed in pancreatic, ovarian, and breast tumors [14–16]. In 2001, Scott et al. showed that* TMPRSS3* was mutated in nonsyndromic autosomal recessive deafness (DFNB8/10), which is associated with both congenital and childhood-onset autosomal recessive deafness [17]. To date, several genetic studies of* TMPRSS3*-related hearing impairment have been conducted, and 31 recessive mutations in* TMPRSS3*, including p.Ala179Thr, which was identified in a Tibetan family from China, were shown to be associated with ARNSHI in more than 14 ethnic groups worldwide [18]. Almost all the mutations were predicted to disrupt the proteolytic activity of the protein [3–5, 8, 10, 11, 17–28]. The hearing impairment in these families was prelingual or postlingual, with a typical ski-slope audiogram configuration. Progressive hearing impairment is another common feature of patients with* TMPRSS3* mutations.

Although hearing impairment in patients with* TMPRSS3* mutations is classified as prelingual or postlingual, the nonrandom distribution pattern of different categories of hearing impairment over the different combinations suggested a correlation between the types of mutation and the onset and severity of hearing impairment, as previously proposed by Weegerink et al. [2]. The differences in the phenotypes in each patient might be the result of the nature of the mutations or location on the gene. This study showed that the combination of p.(Phe13Serfs*∗*12) and p.Ala306Thr results in prelingual, profound hearing impairment in the patient (IV: 1), whereas the combination of p.Arg106Cys and p.Ala306Thr manifests as postlingual, milder hearing impairment in the patient (II: 2, II: 3, and II: 5). Our data suggested that the protein-truncating mutation p.(Phe13Serfs*∗*12) has a severe effect and that p.Arg106Cys is a milder mutation.

The frameshift mutation c.36delC [p.(Phe13Serfs*∗*12)] identified in this study is a novel mutation located within exon 2 which lead to a truncated protein and is close to the reported mutation c.36dupC (p.Phe13fs) [22]. It has been predicted that the deletion of one cytosine will result in a frameshift after amino acid 12 before the transmembrane domain, the addition of 11 novel amino acids, and the premature termination of TMPRSS3. This mutation had CADD score of 33 and was predicted to be damaging.

The missense mutation p.Arg106Cys located within exon 4 causes the negatively charged residue arginine to change to a neutral cysteine at position 106 and is close to the reported mutations p.Asp103Gly and p.Arg109Trp, which are located in the LDLRA domain and are predicted to be involved in the binding of TMPRSS3 with extracellular molecules. Moreover, this mutation introduces a more hydrophobic residue at position 106 and can result in the loss of hydrogen bonds and/or disturb correct folding. This mutation was firstly identified in 1.1% (2/192 alleles) Japanese patients with late-onset hearing loss [29]. Furthermore, it was found in heterozygous form in 25/121312 individuals in the ExAC browser, once in homozygous form. To date, there is no report of the hearing loss caused by the combination of two mild mutations. We speculated that the combination of two mild mutation of* TMPRSS3* such as homozygous p.Arg106Cys would be very subtle or even close to normal.

All affected patients share one common mutation, c.916G>A (p.Ala306Thr), a mutation previously identified in German, Dutch, and Korean deaf patients that substitutes alanine by threonine at amino acid position 306 [2, 5, 8, 23]. Haplotype analysis revealed that p.Ala306Thr is likely to be a founder mutation in the Korean population [8]. This mutation is located in the highly conserved catalytic serine protease domain and probably causes the disturbance of the proteolytic function of TMPRSS3, which may explain the underlying molecular etiology of deafness.

Low-frequency hearing preservation is very useful in auditory rehabilitation. Patient IV: 1 underwent cochlear implantation using the AOS insertion technique, and her residual low-frequency hearing was well preserved, adding to her ability to hear in noisy environments and appreciate the quality of music.

## 5. Conclusions

This study examined the clinical and genetic characteristics of a nonconsanguineous Chinese family (number FH1523) with autosomal recessive hearing loss. Through multiple deafness gene capture and next-generation sequencing, we identified three* TMPRSS3* mutations, c.916G>A, c.36delC, and c.316C>T, as disease-causing mutations. Mutations in* TMPRSS3* have previously been described to cause autosomal recessive progressive hearing impairment with postlingual onset (DFNB8) as well as severe-to-profound prelingual hearing impairment (DFNB10). Interestingly, the hearing impairment of affected individuals in this family varied due to different combinations of mutations. Our results indicated that patients with* TMPRSS3* mutations are good candidates for residual hearing preservation via cochlear implantation. Therefore, screening for* TMPRSS3* in ARNSHI patients with a postlingual, progressive, and ski-slope audiogram is necessary for efficient genetic diagnosis and intervention.

## Supplementary Material

129 deafness genes list includes the information of NCBI Reference, OMIM, Description, CDS bps, # of Exons, Covered Region, inheritance pattern and syndromic or not
