# Supplementary material for: Novel Mutations and Mutation Combinations of TMPRSS3 Cause Various Phenotypes in One Chinese Family with Autosomal Recessive Hearing Impairment
Source: Biomed Res Int. 2017 Jan 29;2017:4707315. doi: 10.1155/2017/4707315 (PMC5303592; doi:10.1155/2017/4707315)
Supplement: Supplementary file 1 — 129 deafness genes list includes the information of NCBI Reference, OMIM, Description, CDS bps, # of Exons, Covered Region, inheritance pattern and syndromic or not [file 4707315.f1.pdf]

Gene	NCBI Reference	OMIM	Description	CDS bps	# of Exons	Covered Region	AD*	AR*	X*	Syn. *
ACTB	NM_001101.3	102630	Actin β	1852	6	CDS only				√
ACTG1	NM_001199954.1	102560	Actin γ 1	2123	6	CDS only	√			
ATP6V1B1	NM_001692.3	192132	Lysosomal ATPase, H+ transporting, V1 subunit B1	1956	14	CDS only				√
ATP6V1B2	NM_001693.3	606939	Lysosomal ATPase, H+ transporting, V1 subunit B2	3054	14	CDS only				√
BCS1L	NM_001079866.1	603647	Ubiquinol-cytochrome c reductase synthesis-like	1454	8	CDS only				√
BSND	NM_057176.2	606412	Bartter syndrome, infantile, with sensorineural deafness	1396	4	CDS only		√		
CATSPER2	NM_001282310.1	607249	Cation channel, sperm associated 2	1755	13	CDS only				√
CCDC50	NM_178335.2	611051	Coiled-coil domain containing 50	8949	12	CDS only	√			
CDH23	NM_022124.5	605516	Cadherin-related 23	11134	70	CDS only		√		
CEACAM16	NM_001039213.2	614591	Carcinoembryonic antigen-related cell adhesion molecule 16	1692	7	CDS only	√			
CLDN14	NM_001146077.1	605608	Claudin 14	1469	3	CDS only		√		
CLRN1	NM_174878.2	606397	Clarin 1	2359	8	CDS only		√		
COCH	NM_001135058.1	603196	Cochlin	2882	11	CDS only	√			
COL11A2	NM_080680.2	120290	Collagen, type XI, α 2	6425	66	CDS only		√		
COL9A2	NM_001852.3	120260	Collagen, type IX, α 2	2831	32	CDS only				√
COL9A3	NM_001853.3	120270	Collagen, type IX, α 3	2485	32	CDS only				√
CRYM	NM_001888.3	123740	Crystallin, mu	1506	10	CDS only	√			
DFNA5	NM_004403.2	608798	Deafness, autosomal dominant 5	2521	10	CDS only	√			
DFNB31	NM_001173425.1	607928	Deafness, autosomal recessive 31 (Whirlin)	4076	12	CDS only		√		
DFNB59	NM_001042702.3	610219	Deafness, autosomal recessive 59	1534	7	CDS only		√		
DIAPH1	NM_005219.4	602121	Diaphanous-related formin 1	5804	28	CDS only	√			
DSPP	NM_014208.3	125485	Dentin sialophosphoprotein	4331	5	CDS only	√			
ECE1	NM_001397.2	600423	Endothelin converting enzyme 1	5114	19	CDS only				√
EDNRA	NM_001957.3	131243	Endothelin receptor type A	4168	8	CDS only				√
EDNRB	NM_000115.3	131244	Endothelin receptor type B	4296	8	CDS only				√
ERCC2	NM_000400.3	126340	Excision repair cross-complementing rodent repair deficiency, complementation group 2	2568	23	CDS only				√
ERCC3	NM_000122.1	133510	Excision repair cross-complementing rodent repair deficiency, complementation group 3	2751	15	CDS only				√
ESPN	NM_031475.2	606351	Espin	3531	13	CDS only		√		
ESRRB	NM_004452.3	602167	Estrogen-related receptor β	3029	11	CDS only		√		
EYA4	NM_004100.4	603550	Eyes absent homolog 4	5697	20	CDS only	√			
FAS	NM_000043.4	134637	Fas cell surface death receptor	2755	9	CDS only				√
FGF3	NM_005247.2	164950	Fibroblast growth factor 3	1548	3	CDS only				√
FGFR3	NM_000142.4	134934	Fibroblast growth factor receptor 3	4304	18	CDS only	√			
FOXI1	NM_012188.4	601093	Forkhead box I1	2296	2	CDS only		√		
GATA3	NM_001002295.1	131320	GATA binding protein 3	3070	6	CDS only				√
GIPC3	NM_133261.2	608792	GIPC PDZ domain containing family, member 3	4317	6	CDS only		√		
GJA1	NM_000165.3	121014	Gap junction protein, α 1	3130	2	CDS only		√		
GJB1	NM_000166.5	304040	Gap junction protein, β 1	1674	2	CDS only				√
GJB2	NM_004004.5	121011	Gap junction protein, β 2	2347	3	Entire gene + 5kb upstream		√		
GJB3	NM_001005752.1	603324	Gap junction protein, β 3	1777	2	CDS only	√			

GJB4	NM_153212.2	605425	Gap junction protein, β 4	2840	2	CDS only				√
GJB6	NM_001110219.2	604418	Gap junction protein, β 6	2178	5	CDS only	√			
GPR98	NM_032119.3	602851	G protein-coupled receptor 98	19333	90	CDS only		√		
GPSM2	NM_013296.4	609245	G-protein signaling modulator 2	3039	15	CDS only		√		
GRHL2	NM_024915.3	608576	Grainyhead-like 2	5231	16	CDS only	√			
GRXCR1	NM_001080476.2	613283	Glutaredoxin, cysteine rich 1	1003	4	CDS only		√		
GSTP1	NM_000852.3	134660	Glutathione S-transferase π 1	986	7	CDS only			√	
HAL	NM_002108.3	609457	Histidine ammonia-lyase	3927	21	CDS only				√
HGF	NM_000601.4	142409	Hepatocyte growth factor	2820	18	CDS only		√		
ILDR1	NM_001199799.1	609739	Immunoglobulin-like domain containing receptor 1	2908	8	CDS only		√		
JAG1	NM_000214.2	601920	Jagged 1	5988	26	CDS only				√
KCNE1	NM_000219.4	176261	Potassium voltage-gated channel, Isk-related family, member 1	3575	4	CDS only				√
KCNJ10	NM_002241.4	602208	Potassium inwardly-rectifying channel, subfamily J, member 10	5323	2	CDS only				√
KCNQ1	NM_000218.2	607542	Potassium voltage-gated channel, KQT-like subfamily, member 1	3262	16	CDS only				√
KCNQ4	NM_004700.3	603537	Potassium voltage-gated channel, KQT-like subfamily, member 4	4116	14	CDS only	√			
KIAA1199	NM_018689.1	608366	KIAA1199	7080	29	CDS only				√
LHFPL5	NM_182548.3	609427	Lipoma HMGIC fusion partner-like 5	2147	4	CDS only		√		
LHX3	NM_014564.3	600577	LIM homeobox 3	2419	6	CDS only				√
LOXHD1	NM_144612.6	613072	Lipoxygenase homology domains 1	6854	40	Chr18: 44152049-44152125		√		
LRTOMT	NM_001145309.3	612414	Leucine rich transmembrane and O-methyltransferase domain containing	3844	9	CDS only		√		
MARVELD2	NM_001038603.2	610572	MARVEL domain containing 2	2297	7	CDS only		√		
mir182	NR_029614.1	611607	microRNA 182	110	1	CDS only				√
mir183	NR_029615.1	611608	microRNA 183	110	1	CDS only				√
mir96	NR_029512.1	611606	microRNA 96	78	1	CDS only	√			
MITF	NM_198159.2	156845	Microphthalmia-associated transcription factor	4815	10	CDS only				√
MSRB3	NM_198080.3	613719	Methionine sulfoxide reductase B3	4307	6	CDS only		√		
MTAP	NM_002451.3	156540	Methylthioadenosine phosphorylase	4937	8	CDS only				√
MT-TD	NC_012920.1	590015	Mitochondrial transfer RNA aspartic acid	68	1	CDS only				√
MT-TH	NC_012920.1	590040	Mitochondrial transfer RNA histidine	69	1	CDS only				√
MT-TI	NC_012920.1	590045	Mitochondrial transfer RNA isoleucine	69	1	CDS only				√
MT-TK	NC_012920.1	590060	Mitochondrial transfer RNA lysine	70	1	CDS only				√
MT-TL1	NC_012920.1	590050	Mitochondrial transfer RNA leucine	75	1	CDS only				√
MT-L2	NC_012920.1	590055	Mitochondrial transfer RNA leucine2	71	1	CDS only				√
MT-TM	NC_012920.1	590065	Mitochondrial transfer RNA methionine	68	1	CDS only				√
MT-TQ	NC_012920.1	590030	Mitochondrial transfer RNA glutamine	72	1	CDS only				√
MT-TS1	NC_012920.1	590080	Mitochondrial transfer RNA serine 1	69	1	CDS only				√
MT-TS2	NC_012920.1	590085	Mitochondrial transfer RNA serine 2	59	1	CDS only				√
MYH14	NM_001145809.1	608568	Myosin, heavy chain 14, non-muscle	6930	43	CDS only	√			
MYH9	NM_002473.4	160775	Myosin, heavy chain 9, non-muscle	7505	41	CDS only	√			
MYO15A	NM_016239.3	602666	Myosin XVA	11876	66	CDS only		√		
MYO1A	NM_001256041.1	601478	Myosin IA	3658	29	CDS only	√			
MYO1C	NM_001080779.1	606538	Myosin IC	4973	32	CDS only				√
MYO1F	NM_012335.3	601480	Myosin IF	4173	28	CDS only				√

MYO3A	NM_017433.4	606808	Myosin IIIA	5798	35	CDS only		√		
MYO6	NM_004999.3	600970	Myosin IV	8662	35	CDS only		√		
MYO7A	NM_000260.3	276903	Myosin 7A	7465	49	CDS only		√		
NDP	NM_000266.3	300658	Norrie disease	2058	3	CDS only				√
NR2F1	XM_005272070.1	132890	Nuclear receptor subfamily 2, group F, member 1	1983	ND	CDS only				√
OTOA	NM_144672.3	607038	Otoancorin	3624	28	CDS only		√		
OTOF	NM_194248.2	603681	Otoferlin	7171	47	CDS only		√		
OTOR	NM_020157.3	606067	Otoraplin	1482	4	CDS only				√
P2RX2	NM_170683.3	600844	Purinergic receptor P2X, ligand-gated ion channel, 2	1945	10	CDS only	√			
PAX3	NM_181457.3	606597	Paired box 3	2032	8	CDS only				√
PCDH15	NM_001142763.1	605514	Protocadherin-related 15	7042	33	CDS only		√		
PDZD7	NM_001195263.1	612971	PDZ domain-containing 7	4164	17	CDS only				√
PMP22	NM_000304.3	601097	Peripheral myelin protein 22	1861	5	CDS only				√
POU3F4	NM_000307.4	300039	POU class 3 homeobox 4	1507	1	CDS only			√	
POU4F3	NM_002700.2	602460	POU class 4 homeobox 3	1182	2	CDS only	√			
PRPS1	NM_002764.3	311850	Phosphoribosyl pyrophosphate synthetase 1	2156	7	CDS only			√	
PTPRQ	NM_001145026.1	603317	Protein tyrosine phosphatase, receptor type, Q	8066	3	Chr12: 80849273-80849842; 80878212-80878383		√		
RDX	NM_001260492.1	179410	Radixin	2761	14	CDS only		√		
SERPINB6	NM_004568.5	173321	Serpin peptidase inhibitor, clade B (ovalbumin), member 6	1932	7	CDS only		√		
SIX1	NM_005982.3	601205	SIX homeobox 1	2687	2	CDS only	√			
SIX5	NM_175875.4	600963	SIX homeobox 5	3352	3	CDS only				√
SLC17A8	NM_139319.2	607557	Solute carrier family 17 (vesicular glutamate transporter), member 8	3983	12	CDS only	√			
SLC26A4	NM_000441.1	605646	Solute carrier family 26 (anion exchanger), member 4	4930	21	CDS only		√		
SLC26A5	NM_198999.2	604943	Solute carrier family 26 (anion exchanger), member 5	2697	20	CDS only		√		
SLC4A11	NM_001174090.1	610206	Solute carrier family 4, sodium borate transporter, member 11	3268	20	CDS only				√
SMPX	NM_014332.2	300226	Small muscle protein, X-linked	951	5	CDS only			√	
SNAI2	NM_003068.4	602150	Snail family zinc finger 2	2112	3	CDS only				√
SOX2	NM_003106.3	184429	SRY (sex determining region Y)-box 2	2520	1	CDS only				√
SPINK5	NM_001127698.1	605010	Serine peptidase inhibitor, Kazal type 5	3745	34	CDS only				√
STRC	NM_153700.2	606440	Stereocilin	5515	29	CDS only		√		
TBL1X	NM_005647.3	300196	Transducin (β)-like 1X-linked	5715	18	CDS only				√
TCF21	NM_198392.2	603306	Transcription factor 21	3249	3	CDS only				√
TECTA	NM_005422.2	602574	Tectorin α	6468	23	CDS only		√		
TFCP2	NM_005653.4	189889	Transcription factor CP2	3715	15	CDS only				√
TIMM8A	NM_004085.3	300356	Translocase of inner mitochondrial membrane 8 homolog A	1459	2	CDS only			√	
TJP2	NM_004817.3	607709	Tight junction protein 2	4725	23	CDS only	√			
TMC1	NM_138691.2	606706	Transmembrane channel-like 1	3201	24	CDS only		√		
TMIE	NM_147196.2	607237	Transmembrane inner ear	1861	4	CDS only		√		
TMPRSS3	NM_024022.2	605511	Transmembrane protease, serine 3	2463	13	CDS only		√		
TMPRSS5	NM_030770.2	606751	Transmembrane protease, serine 5	2233	13	CDS only				√
TPRN	NM_001128228.2	613354	Taperin	2641	4	CDS only		√		

TRIOBP	NM_001039141.2	609761	TRIO and F-actin binding protein	10159	24	CDS only		√
USH1C	NM_153676.3	605242	Usher syndrom 1C	3246	28	CDS only		√
USH1G	NM_173477.3	607696	Usher syndrome 1G	3568	3	CDS only		√
USH2A	NM_206933.2	608400	Usher syndrome 2A	18883	72	CDS only		√
WFS1	NM_006005.3	606201	Wolfram syndrome 1	3640	8	CDS only	√	

* Inheritance pattern: AD = autosomal dominant
AR = autosomal recessive
X = X-linked
Syn = syndromic deafness

Otogenetics Corporation
400 Pinnacle Way, Suite 435
Norcross, GA 30071 USA
